# Common juniper, the oldest nonclonal woody species across the tundra biome and the European continent

**DOI:** 10.1002/ecy.4514

**Published:** 2025-01-21

**Authors:** Marco Carrer, Raffaella Dibona, Davide Frigo, Ludmila Gorlanova, Rashit Hantemirov, Lucrezia Unterholzner, Signe Normand, Urs Albert Treier, Angela Luisa Prendin

**Affiliations:** ^1^ Department of Land Environment Agriculture and Forestry University of Padova Legnaro Italy; ^2^ Ural Division of the Russian Academy of Sciences Institute of Plant and Animal Ecology Ekaterinburg Russia; ^3^ Ural Institute for Humanities Ural Federal University Ekaterinburg Russia; ^4^ Technische Universität Dresden, Chair of Forest Growth and Woody Biomass Production Tharandt Germany; ^5^ Department of Biology Section for Ecoinformatic and Biodiversity, Aarhus University Aarhus Denmark

**Keywords:** dendrochronology, *Juniperus communis* L., nature conservation, old growth, old shrubs, plant age, ring width, tundra

One of the most remarkable characteristics of trees, alongside their size, is their longevity. Trees frequently live for several centuries and even well over a thousand years for a limited group of taxa. The number of centennial‐ or millennial‐old woody species is steadily increasing due to continuous discoveries mostly associated with the growing efforts and attention devoted to preserving and studying long‐lived individuals (Brown, 1996, 2024). When present, these ancient organisms represent a slowly emerging property in vegetation assemblages, strictly tied to the natural and anthropogenic disturbance history of the ecosystem in which they reside. Given that the presence or replacement of very old woody individuals, ancient woodlands, and primary forests cannot be restored without a significant passage of time, there is an increasing emphasis on recognizing, studying, and protecting them. Manifold are indeed the positive benefits that old woody plants provide: they can be considered hotspots for biodiversity within the ecosystem, promoting the recovery process after disturbance as biological legacies. They also stand as important witnesses to past climate variability, enduring hundreds or thousands of years encompassing warm, wet, dry, or cold phases, along with a multitude of extreme weather events. Finally, due to their extended residence time, old woody plants significantly contribute to increasing and maintaining carbon storage within the ecosystem (Gilhen‐Baker et al., 2022), while forest stands hosting old trees act as substantial sinks within the global carbon cycle (Luyssaert et al., 2008), although their impact may be less than previously estimated (Gundersen et al., 2021).

Nevertheless, within woody plants, the potential to attain extended lifespans is not exclusive to trees. Over the last few years, an increasing body of evidence has shown that even shrubs can endure for centuries. Several reports document the discovery of exceptionally old shrub individuals across a broad range of taxa and environments, from the Tibetan Plateau (Lu et al., 2015) to the Mediterranean (Mathaux et al., 2016) and from the high latitudes (Hallinger et al., 2010; Hantemirov et al., 2000) to the high elevations in the Alps (Carrer et al., 2023; Francon et al., 2017). However, despite their lower stature, shrub communities hold inestimable ecological value and should be considered as important as trees. They usually thrive in extreme environmental conditions. With their prostrate growth habit, shrubs can extend their presence far beyond the latitudinal and elevational limits of trees, acting as the outposts of woody plants from the warm and xeric Mediterranean to cold tundra regions. For this reason, ongoing climate change is likely to induce remarkable consequences in shrub communities, leading to either a reduction or an expansion of their range. Recent hotter and more severe droughts are inducing diffuse tree mortality (Allen et al., 2010), potentially leading to shrub replacement. In parallel, in the Arctic, high‐latitude, and alpine tundra ecosystems, many investigations have documented a positive shift in shrub abundance (Myers‐Smith et al., 2011). However, in heat‐limited environments, current warming not only relaxes previous constraints for shrubs but also creates improved growing conditions that benefit trees. This leads to their expansion beyond the former treeline, increased stand density, and growth rate, which, in turn, may suppress and outcompete shrubs (Berner & Goetz, 2022). The interspersing of positive and negative responses and dynamics of woody tundra vegetation to climate change adds complexity to understanding the observed greening and browning trends in the Arctic (Myers‐Smith et al., 2020).

We conducted multiple field campaigns across Arctic and subarctic tundra, from Greenland to the Polar Urals, selecting sites at regional level where the target species *Juniperus communis* L. was common. This allows for the collection of a significant number of samples. All the sites were located mostly above the treeline, when present. We also ensured the homogeneity of the sampling area in terms of environmental settings, including slope, aspect, soil features, and vegetation. Additionally, we avoided areas characterized by only scattered juniper individuals or clearly affected by natural disturbances such as herbivory, fire, and landslides. At each site, we selected both living and dead shrubs (Figure 1), focusing on the oldest‐looking individuals, and then saw‐cut a basal disk from one of the main prostrate stems. Our sampling strategy mirrored that applied in classical dendrochronology (Pilcher, 1990). Therefore, it is crucial to note that in this way, the age structure of the sampled populations, especially those of younger individuals, cannot be reliably inferred. However, we are confident that our approach offers a reliable depiction of the maximum age that common junipers can reach at each site. Considering the wide distribution of the species, we acknowledge the very likely presence of even older individuals in the vast tundra biome.

**FIGURE 1:**
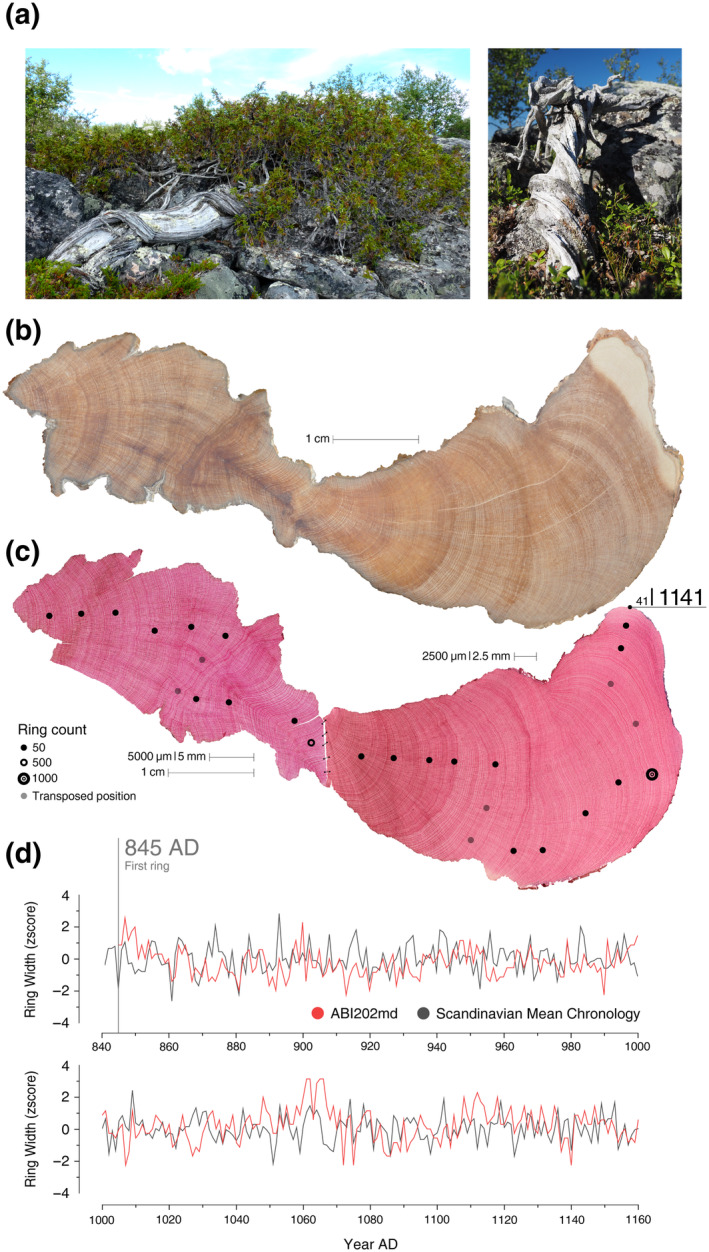
(A) Typical growth habit of common juniper in the tundra environment (photo credit: Lucrezia Unterholzner and Marco Carrer). (B) Section of a branch from the oldest living sample collected in northern Sweden (photo credit: Marco Carrer). (C) Stained microsections from the same sample used to precisely detect the yearly growth rings. Dots on the image provide the marks obtained through the ring count. This sample has 1141 visible rings. The high‐resolution images of this picture are available in Prendin et al. (2024). (D) By collating all the cross‐dated samples from the two northern Scandinavian sites (Abisko [SWE] and Kevo [FIN]), we built a mean chronology that permits us to absolutely date this sample. The two plots show the first 320 measured years (cross‐dating statistics: GLK = 0.59; Corr. = 0.38; *t*‐value = 7.1); the first ring dates back to 845 AD. Therefore, this sample is 1176 years old, consisting of 1141 visible rings plus 35 missing rings.

In the laboratory, the disks were sanded with progressively finer grit sandpaper for a clear visualization of the annual rings. In some cases, due to the extremely slow growth rate and reduced dimensions of certain individuals, we applied microscopic sample preparation to enhance the visibility of the ring‐width sequences (von Arx et al., 2016) (Figure 1). Juniper stems under limiting conditions are typically irregular and characterized by marked asymmetrical cambial activity and missing rings. For a reliable and effective representation of the ring‐width series, we measured one to four radii per sample, following lines without exceptionally narrow or wedging rings (Carrer et al., 2019) (Figure 1).

Age determination was established by cross‐dating the ring‐width measurements, which involves matching ring‐width patterns between different measurement series to ensure that each ring is accurately assigned its calendar year of formation. Cross‐dating is a hierarchical process that involves visually comparing single measurement lines within individuals, considering the mean individual series between individuals of the same population and finally statistically verifying dating and measurement errors (Grissino‐Mayer, 2001). In this phase, we paid special attention to the detection of missing rings, which are typically common in junipers and are not solely related to extreme climate or disturbance events. It is important to emphasize that the age we established represents the cross‐dated age at the sampling point where we collected the stem disk. We also did not add years in relation to any missing part or pith offset. Therefore, the shrub from which we collected the disk is almost certainly older (with an estimation of 10–200 years) than what we defined.

Sitewide, common juniper consistently represents the longest lived woody species ever documented at the regional level within the respective tundra vegetation assemblage (Figure 2, Appendix S1: Table S1). We encountered numerous individuals older than 300 years, with some exceeding 500 years. Notably, at two sites in northern Fennoscandia, several common junipers surpassed a thousand years in age, with the oldest being 1647 years old (Figure 2). This discovery establishes the oldest dendrochronologically dated nonclonal woody plant on the European continent and the world's oldest shrub to date. Nevertheless, even in cases where ages are not remarkable—possibly due to significant disturbance pressure, as observed in the Faroe Islands or Iceland—common juniper continues to stand as the oldest recorded plant to date on these islands.

**FIGURE 2:**
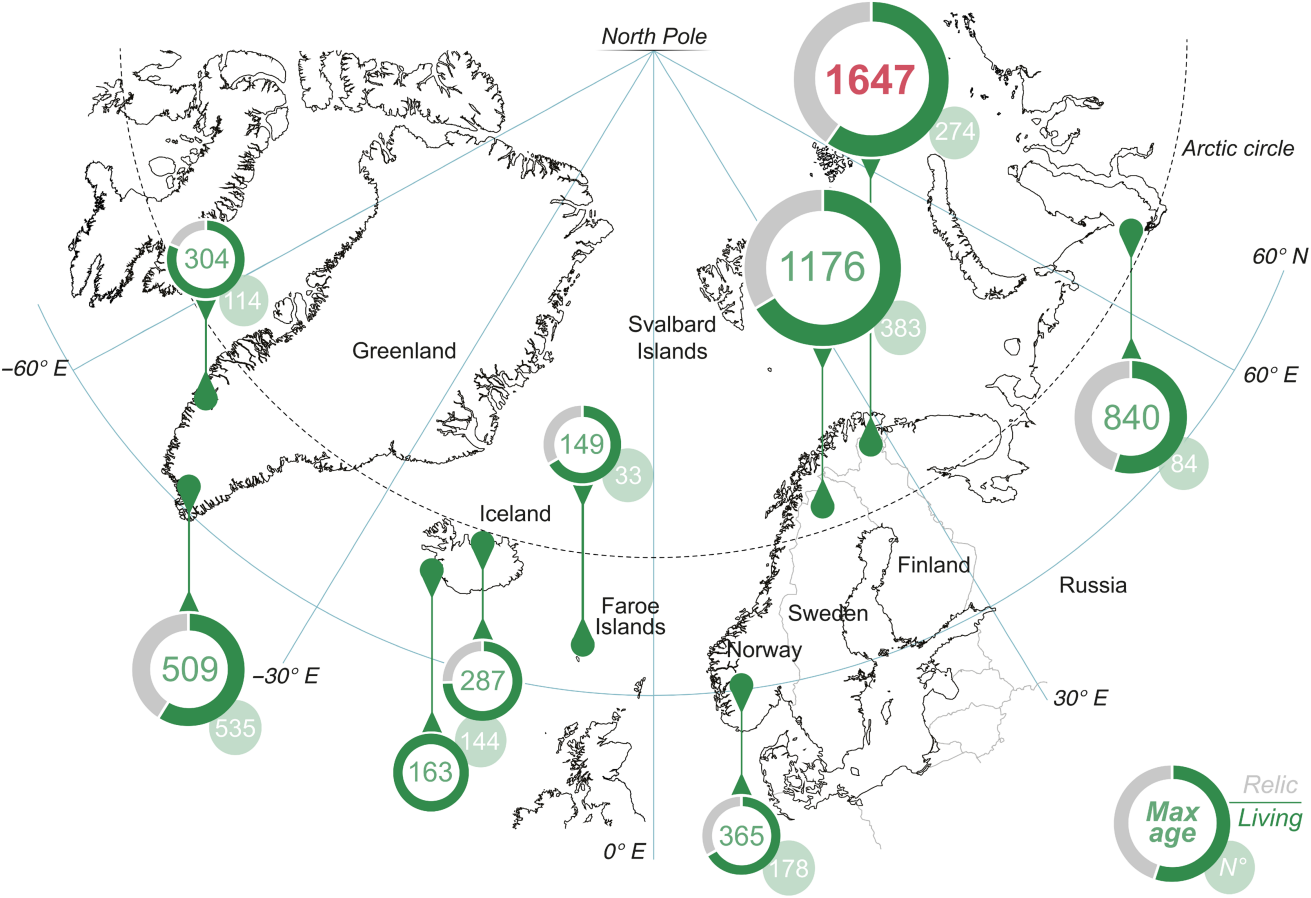
Common juniper ages across the tundra biome. The numbers represent the maximum juniper age and the number of individuals sampled at each site. Pie charts show the percentages of living (green) and dead (gray) individuals collected.

One of the most distinctive representatives of woody plants within the tundra biome is the common juniper. It is the most widely distributed conifer species in the world, with a circumpolar range that spans from well beyond the polar circle in Alaska, Scandinavia, and Siberia to the Himalayas and Atlas Mountains in northern Africa (Caudullo et al., 2017; Farjon, 2010). Characterized by prominent polymorphisms, habitat factors, mostly related to climate (temperature, precipitation, and snow cover permanence), can dramatically affect plant growth, which can range from creeping to erect. Accordingly, within‐population variability can be of the same magnitude as that between recognized infraspecific taxa (Farjon, 2010). Common juniper possesses additional key characteristics that make it attractive for investigations of environmental and vegetation dynamics. In particular, it is a long‐living organism and, like many conifers, exhibits distinct and usually clearly detectable ring widths, making it a suitable candidate for effective dendrochronological analysis. However, despite being one of the most peculiar species within the vast tundra biome, common juniper remains largely overlooked in the scientific community, especially in retrospective investigations. This was likely due to the challenges in processing and measuring the samples, as well as the relatively lower cross‐dating potential, which requires a much larger sample size to obtain a sufficient number of correctly dated samples for reliable inferences.

Tundra junipers exhibit low growth rates and irregular growth habit, often associated with marked asymmetric cambial activity and strip‐bark stems (Figure 1). They endure in extreme environments where resources are scarce (Schulman, 1954). Like ancient trees, isolated and stunted old tundra junipers are relatively free from competition and rarely experience severe disturbances (Piovesan et al., 2020). This species, therefore, supports the growth–longevity trade‐off pattern observed in the evolution and expression of life history traits in many tree species, especially gymnosperm, across various biomes (Liu et al., 2022; Piovesan et al., 2020; Piovesan & Biondi, 2021), suggesting that this pattern likely applies even to prostrate shrubs. Nonetheless, its growing rings are not only visible, as mentioned earlier but also cross‐datable, making them a valuable and powerful proxy for retrospective, annually resolved environmental (Frigo et al., 2023; Tumajer et al., 2021) and climate analysis (Carrer et al., 2023) or as a dating tool to pinpoint the age of archaeological sites or woody artifacts (e.g., the Tupilaqs) in treeless regions (Appendix S1: Figure S1). Due to cross‐dating and the inclusion of relic samples, it is also possible to extend the ring‐width information further back in time, well beyond the limits set by living shrubs (Appendix S1: Figure S2, Table S2). However, we must highlight that the sampling process is usually more destructive compared with the classical dendrochronological approach, which may limit its use in extensive research. Given the small dimensions and the common strip‐bark growth of the specimens, coring is not feasible, making disk cutting the only viable alternative.

A remarkable age, characterized by the presence of old individuals, is likely the most distinctive trait of old‐growth forests. The scientific community has recognized the high value of such ecosystems in relation to the broad spectrum of services they provide. The same perspective, despite the much smaller size in terms of canopy height and biomass, should be adopted for old‐growth shrub communities. Tundra ecosystems with long‐lived woody species such as common junipers are typically not under the pressure of land use change or deforestation, unlike several old‐growth forests around the globe. Nevertheless, current warming at high latitudes, along with consequent natural vegetation dynamics and climate change‐driven increases in the intensity and frequency of natural disturbances (e.g., wildfires), may pose even more serious threats. The northward advance of forest vegetation can rapidly outcompete old junipers and alter the natural disturbance regime that commonly shapes tundra assemblages. This emphasizes the need to first recognize and then implement conservation efforts to prevent the loss of this unique component of the tundra biome.

## CONFLICT OF INTEREST STATEMENT

The authors declare no conflicts of interest.

## Supporting information


Appendix S1.

## Data Availability

Data (Prendin et al., 2024) are available in Zenodo at https://doi.org/10.5281/zenodo.11307027.

## References

[ecy4514-bib-0001] Allen, C. D. , A. K. Macalady , H. Chenchouni , D. Bachelet , N. McDowell , M. Vennetier , T. Kitzberger , A. Rigling , D. D. Breshears , and E. Hogg . 2010. “A Global Overview of Drought and Heat‐Induced Tree Mortality Reveals Emerging Climate Change Risks for Forests.” Forest Ecology and Management 259: 660–684.

[ecy4514-bib-0002] Berner, L. T. , and S. J. Goetz . 2022. “Satellite Observations Document Trends Consistent With a Boreal Forest Biome Shift.” Global Change Biology 28: 3275–3292. 35199413 10.1111/gcb.16121 PMC9303657

[ecy4514-bib-0003] Brown, P. M. 1996. “OLDLIST: A Database of Maximum Tree Ages.” In Proceedings of the International Conference on Tree Rings, Environment, and Humanity: Relationships and Processes, Tucson, Arizona. 727–731.

[ecy4514-bib-0004] Brown, P. M. 2024. “OLDLIST, a Database of Old Trees.” Rocky Mountain Tree‐Ring Research, Fort Collins, CO. http://www.rmtrr.org/oldlist.htm.

[ecy4514-bib-0005] Carrer, M. , R. Dibona , A. L. Prendin , and M. Brunetti . 2023. “Recent Waning Snowpack in the Alps Is Unprecedented in the Last Six Centuries.” Nature Climate Change 13: 155–160.

[ecy4514-bib-0006] Carrer, M. , E. Pellizzari , A. L. Prendin , M. Pividori , and M. Brunetti . 2019. “Winter Precipitation—Not Summer Temperature—Is Still the Main Driver for Alpine Shrub Growth.” Science of the Total Environment 682: 171–179. 31112818 10.1016/j.scitotenv.2019.05.152

[ecy4514-bib-0007] Caudullo, G. , E. Welk , and J. San‐Miguel‐Ayanz . 2017. “Chorological Maps for the Main European Woody Species.” Data in Brief 12: 662–666. 28560272 10.1016/j.dib.2017.05.007 PMC5435575

[ecy4514-bib-0008] Farjon, A. 2010. A Handbook of the World's Conifers, Vol. 2. Leiden: Brill.

[ecy4514-bib-0009] Francon, L. , C. Corona , E. Roussel , J. Lopez Saez , and M. Stoffel . 2017. “Warm Summers and Moderate Winter Precipitation Boost *Rhododendron ferrugineum* L. Growth in the *Taillefer massif* (French Alps).” Science of the Total Environment 586: 1020–1031. 28214115 10.1016/j.scitotenv.2017.02.083

[ecy4514-bib-0010] Frigo, D. , Ó. Eggertsson , A. L. Prendin , R. Dibona , L. Unterholzner , and M. Carrer . 2023. “Growth Form and Leaf Habit Drive Contrasting Effects of Arctic Amplification in Long‐Lived Woody Species.” Global Change Biology 29: 5896–5907. 37526296 10.1111/gcb.16895

[ecy4514-bib-0011] Gilhen‐Baker, M. , V. Roviello , D. Beresford‐Kroeger , and G. N. Roviello . 2022. “Old Growth Forests and Large Old Trees as Critical Organisms Connecting Ecosystems and Human Health. A Review.” Environmental Chemistry Letters 20: 1529–1538. 35002589 10.1007/s10311-021-01372-y PMC8728480

[ecy4514-bib-0012] Grissino‐Mayer, H. D. 2001. “Evaluating Crossdating Accuracy: A Manual and Tutorial for the Computer Program COFECHA.” Tree‐Ring Research 57: 205–221.

[ecy4514-bib-0013] Gundersen, P. , E. E. Thybring , T. Nord‐Larsen , L. Vesterdal , K. J. Nadelhoffer , and V. K. Johannsen . 2021. “Old‐Growth Forest Carbon Sinks Overestimated.” Nature 591: E21–E23. 33762763 10.1038/s41586-021-03266-z

[ecy4514-bib-0014] Hallinger, M. , M. Manthey , and M. Wilmking . 2010. “Establishing a Missing Link: Warm Summers and Winter Snow Cover Promote Shrub Expansion into Alpine Tundra in Scandinavia.” New Phytologist 186: 890–899. 20345642 10.1111/j.1469-8137.2010.03223.x

[ecy4514-bib-0015] Hantemirov, R. M. , L. A. Gorlanova , and S. G. Shiyatov . 2000. “Pathological Tree‐Ring Structures in Siberian Juniper (*Juniperus sibirica* Burgsd.) and their Use for Reconstructing Extreme Climatic Events.” Russian Journal of Ecology 31: 167–173.

[ecy4514-bib-0016] Liu, J. , S. Xia , D. Zeng , C. Liu , Y. Li , W. Yang , B. Yang , J. Zhang , F. Slik , and D. B. Lindenmayer . 2022. “Age and Spatial Distribution of the World's Oldest Trees.” Conservation Biology 36: e13907. 10.1111/cobi.13907 35288993

[ecy4514-bib-0017] Lu, X. , J. J. Camarero , Y. Wang , E. Liang , and D. Eckstein . 2015. “Up to 400‐Year‐Old *Rhododendron* Shrubs on the Southeastern Tibetan Plateau: Prospects for Shrub‐Based Dendrochronology.” Boreas 44: 760–768.

[ecy4514-bib-0018] Luyssaert, S. , E. D. Schulze , A. Börner , A. Knohl , D. Hessenmöller , B. E. Law , P. Ciais , and J. Grace . 2008. “Old‐Growth Forests as Global Carbon Sinks.” Nature 455: 213–215. 18784722 10.1038/nature07276

[ecy4514-bib-0019] Mathaux, C. , J.‐P. Mandin , C. Oberlin , J.‐L. Edouard , T. Gauquelin , and F. Guibal . 2016. “Ancient Juniper Trees Growing on Cliffs: Toward a Long Mediterranean Tree‐Ring Chronology.” Dendrochronologia 37: 79–88.

[ecy4514-bib-0020] Myers‐Smith, I. H. , B. C. Forbes , M. Wilmking , M. Hallinger , T. Lantz , D. Blok , K. D. Tape , et al. 2011. “Shrub Expansion in Tundra Ecosystems: Dynamics, Impacts and Research Priorities.” Environmental Research Letters 6: 045509.

[ecy4514-bib-0021] Myers‐Smith, I. H. , J. T. Kerby , G. K. Phoenix , J. W. Bjerke , H. E. Epstein , J. J. Assmann , C. John , et al. 2020. “Complexity Revealed in the Greening of the Arctic.” Nature Climate Change 10: 106–117.

[ecy4514-bib-0022] Pilcher, J. R. 1990. “Sample Preparation, Cross‐Dating, and Measurement.” In Methods of Dendrochronology, edited by E. R. Cook and L. A. Kairiukstis , 40–51. Dordrecht: Kluwer Academic Publishers.

[ecy4514-bib-0023] Piovesan, G. , M. Baliva , L. Calcagnile , M. D'Elia , I. Dorado‐Liñán , J. Palli , A. Siclari , and G. Quarta . 2020. “Radiocarbon Dating of Aspromonte Sessile Oaks Reveals the Oldest Dated Temperate Flowering Tree in the World.” Ecology 101: 1–4. 10.1002/ecy.3179 32860441

[ecy4514-bib-0024] Piovesan, G. , and F. Biondi . 2021. “On Tree Longevity.” New Phytologist 231: 1318–1337. 33305422 10.1111/nph.17148

[ecy4514-bib-0029] Schulman, E. 1954. “Longevity Under Adversity in Conifers.” Science 119: 396–399. 17842727 10.1126/science.119.3091.396

[ecy4514-bib-0026] Prendin, A. L. , M. Carrer , D. Frigo , and G. Fanchin . 2024. “High Resolution Microsection Images for: Common Juniper, the Oldest Living Non‐Clonal Woody Species Across the Tundra Biome and the European Continent.” Zenodo. 10.5281/zenodo.11307027. PMC11751590 39838703

[ecy4514-bib-0027] Tumajer, J. , A. Buras , J. J. Camarero , M. Carrer , R. Shetti , M. Wilmking , J. Altman , G. Sangüesa‐Barreda , and J. Lehejček . 2021. “Growing Faster, Longer or Both? Modelling Plastic Response of *Juniperus communis* Growth Phenology to Climate Change.” Global Ecology and Biogeography 30: 2229–2244.

[ecy4514-bib-0028] von Arx, G. , A. Crivellaro , A. L. Prendin , K. Cufar , and M. Carrer . 2016. “Quantitative Wood Anatomy‐Practical Guidelines.” Frontiers in Plant Science 7: 781. 27375641 10.3389/fpls.2016.00781 PMC4891576

