# Supplementary material for: Common juniper, the oldest nonclonal woody species across the tundra biome and the European continent
Source: Ecology. 2025 Jan 21;106(1):e4514. doi: 10.1002/ecy.4514 (PMC11751590; doi:10.1002/ecy.4514)
Supplement: Supplementary file 1 — Appendix S1. [file ECY-106-e4514-s001.pdf]

***Journal*: ECOLOGY**

***Title*: Common juniper, the oldest non-clonal woody species across the tundra biome and the European continent**

Authors: Marco Carrer, Raffaella Dibona, Davide Frigo, Ludmila Gorlanova, Rashit Hantemirov, Lucrezia Unterholzner, Signe Normand, Urs Albert Treier, Angela Luisa Prendin

Appendix S1: Tables and Figures

Table S1. The oldest (more than 100 years old) documented common junipers and other non-clonal tundra shrub taxa.

Species	Number of years	Dating method	Location	Reference
Loiseleuria procumbens	110	Ring count	Central Europe	(Schweingruber and Poschlod 2005)
Alnus viridis	111	Crossdating	Laborovaya, Russia	(Macias-Fauria et al. 2012)
Arctostaphylos alpinus	117	Ring count	Ittoqqortoormiit, Greenland	(Büntgen et al. 2015)
Rhododendrum hirsutum	132	Ring count	Central Europe	(Schweingruber and Poschlod 2005)
Alnus viridis ssp. fruticosa	142	Crossdating	Sagavanirktok River, Alaska	(Drew et al. 2023)
Dryas octopetala	156	Ring count	Ittoqqortoormiit, Greenland	(Büntgen et al. 2015)
Betula nana	162	Ring count	Ittoqqortoormiit, Greenland	(Büntgen et al. 2015)
Cassiope tetragona	183	Crossdating	Endalen, Svalbard	(Weijers et al. 2010)
Juniperus communis	192	Ring count	Upper Teesdale, UK	(Gilbert 1980)
Rhododendron ferrugineum	202	Ring count	Central Europe	(Schweingruber and Poschlod 2005)
Rhododendron lapponicum	204	Ring count	Ittoqqortoormiit, Greenland	(Büntgen et al. 2015)
Salix arctica	270	Crossdating	Ellesmere Island, Canada	(Boulanger-Lapointe et al. 2014)
Juniper pingii	324	Crossdating	Nam Co, Tibet, China	(Liang et al. 2011)
Juniperus nana	334	Crossdating	Abisko, Sweden	(Hallinger et al. 2010)
Juniperus communis spp. nana	352	Ring count	Central Europe	(Schweingruber and Poschlod 2005)
Rhododendron aganniphum var. schizopeplum	401	Crossdating	Sygera Mountains, Tibet, China	(Lu et al. 2015)
Juniperus communis	401	Crossdating	Val Ventina, Italy	(Pellizzari et al. 2014)
Juniperus communis	556	Crossdating	Keivy, Kola Peninsula, Russia	(Shumilov et al. 2007)
Juniperus communis	683	Ring count	Gardsjøen Lake, Norway	(Lehejček et al. 2024)

Table S1 References

Boulanger-Lapointe, N., E. Lévesque, S. Boudreau, G. H. R. Henry, and N. M. Schmidt. 2014. Population structure and dynamics of Arctic willow (*Salix arctica*) in the High Arctic. Journal of Biogeography 41:1967-1978.

Büntgen, U., L. Hellmann, W. Tegel, S. Normand, I. Myers-Smith, A. V. Kirdyanov, D. Nievergelt, and F. H. Schweingruber. 2015. Temperature-induced recruitment pulses of Arctic dwarf shrub communities. Journal of Ecology 103:489-501.

Drew, J. W., M. S. Bret-Harte, A. Buchwal, and C. Heslop. 2023. Age matters: Older *Alnus viridis* ssp. *fruticosa* are more sensitive to summer temperatures in the Alaskan Arctic. Functional Ecology 37:1463-1475.

Gilbert, O. L. 1980. Juniper in Upper Teesdale. Journal of Ecology 68:1013-1024.

Hallinger, M., M. Manthey, and M. Wilmking. 2010. Establishing a missing link: warm summers and winter snow cover promote shrub expansion into alpine tundra in Scandinavia. New Phytologist 186:890-899.

Lehejček, J., M. Roman, M. Lexa, E. P. Aspholm, and J. Mašek. 2024. Old Juniper Troll stand - The oldest shrub population from Scandinavia. Journal of Forest Science 70:176-184.

Liang, E., X. Lu, P. Ren, X. Li, L. Zhu, and D. Eckstein. 2011. Annual increments of juniper dwarf shrubs above the tree line on the central Tibetan Plateau: a useful climatic proxy. Annals of Botany 109:721-728.

Lu, X., J. J. Camarero, Y. Wang, E. Liang, and D. Eckstein. 2015. Up to 400-year-old *Rhododendron* shrubs on the southeastern Tibetan Plateau: prospects for shrub-based dendrochronology. Boreas 44:760-768.

Macias-Fauria, M., B. C. Forbes, P. Zetterberg, and T. Kumpula. 2012. Eurasian Arctic greening reveals teleconnections and the potential for structurally novel ecosystems. Nature Climate Change 2:613-618.

Pellizzari, E., M. Pividori, and M. Carrer. 2014. Winter precipitation effect in a mid-latitude temperature-limited environment: the case of common juniper at high elevation in the Alps. Environmental Research Letters 9:104021.

Schweingruber, F. H., and P. Poschlod. 2005. Growth rings in herbs and shrubs: life span, age determination and stem anatomy. Swiss Federal Research Institute WSL Birmensdorf, Switzerland.

Shumilov, O. I., E. A. Kasatkina, N. V. Lukina, I. Y. Kirtsideli, and A. G. Kanatjev. 2007. Paleoclimatic potential of the northernmost juniper trees in Europe. Dendrochronologia 24:123-130.

Weijers, S., R. Broekman, and J. Rozema. 2010. Dendrochronology in the High Arctic: July air temperatures reconstructed from annual shoot length growth of the circumarctic dwarf shrub *Cassiope tetragona*. Quaternary Science Reviews 29:3831-3842.

Fig. S1. The promising application of common juniper ring width measurements in treeless regions. A) A woman's face, carved on a juniper branch by an Inuit in Greenland (photo credit Marco Carrer). B) The base of the artifact where we measured 324 rings (photo credit Marco Carrer). C) By comparing the mean ring-width chronology built for the Narsarsuaq site with the measurements obtained from the artifact, it was possible to date the cutting year of the juniper branch back to 1961 AD (crossdating statistics: GLK = 0.67; Corr. = 0.43; t-value = 8.4).

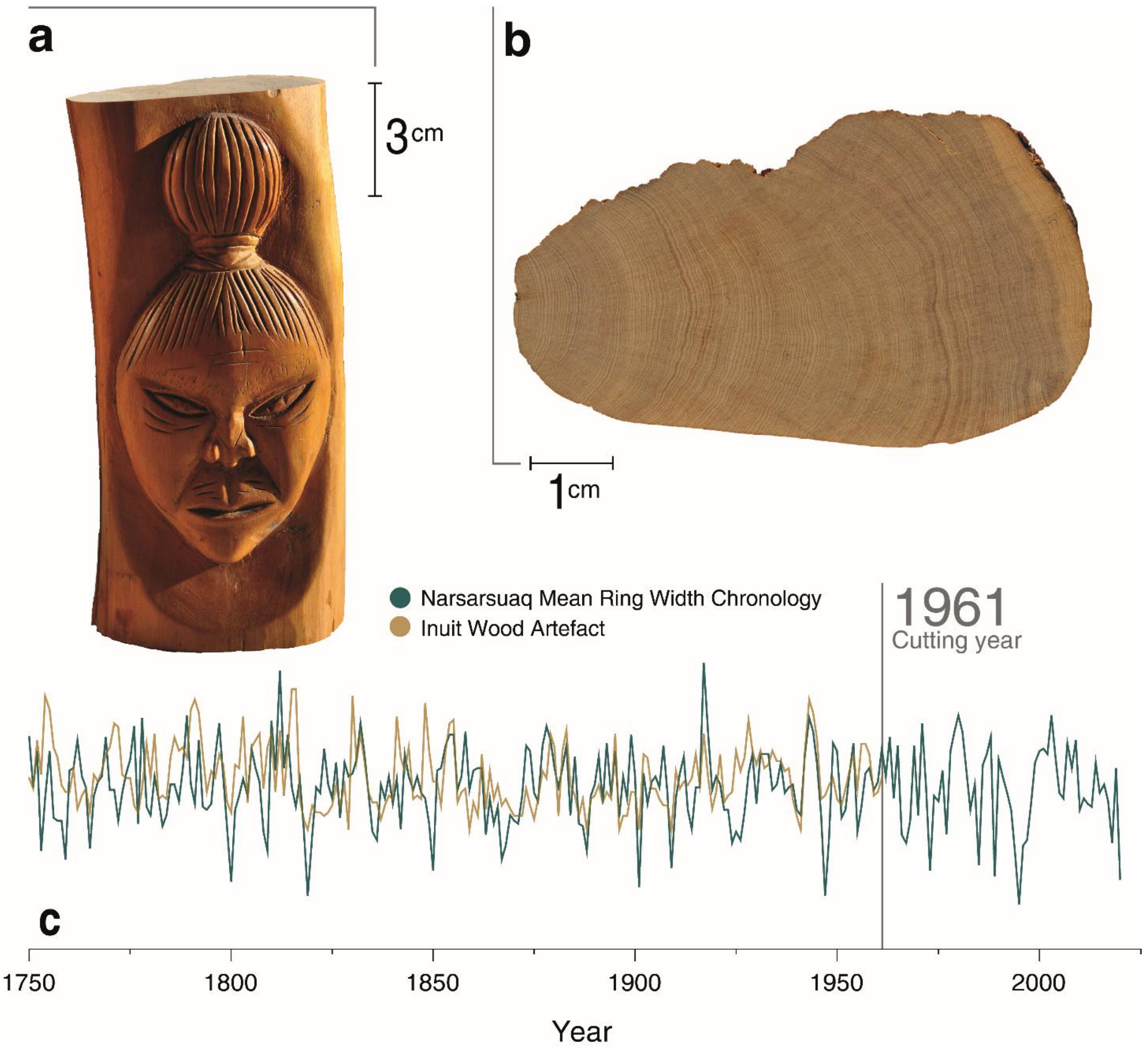

Fig. S2. Raw mean ring-width chronology for each sampling site computed using the crossdated individual series (dark green line), together with the standard deviation (light green band) and the yearly sample depth (yellow area).

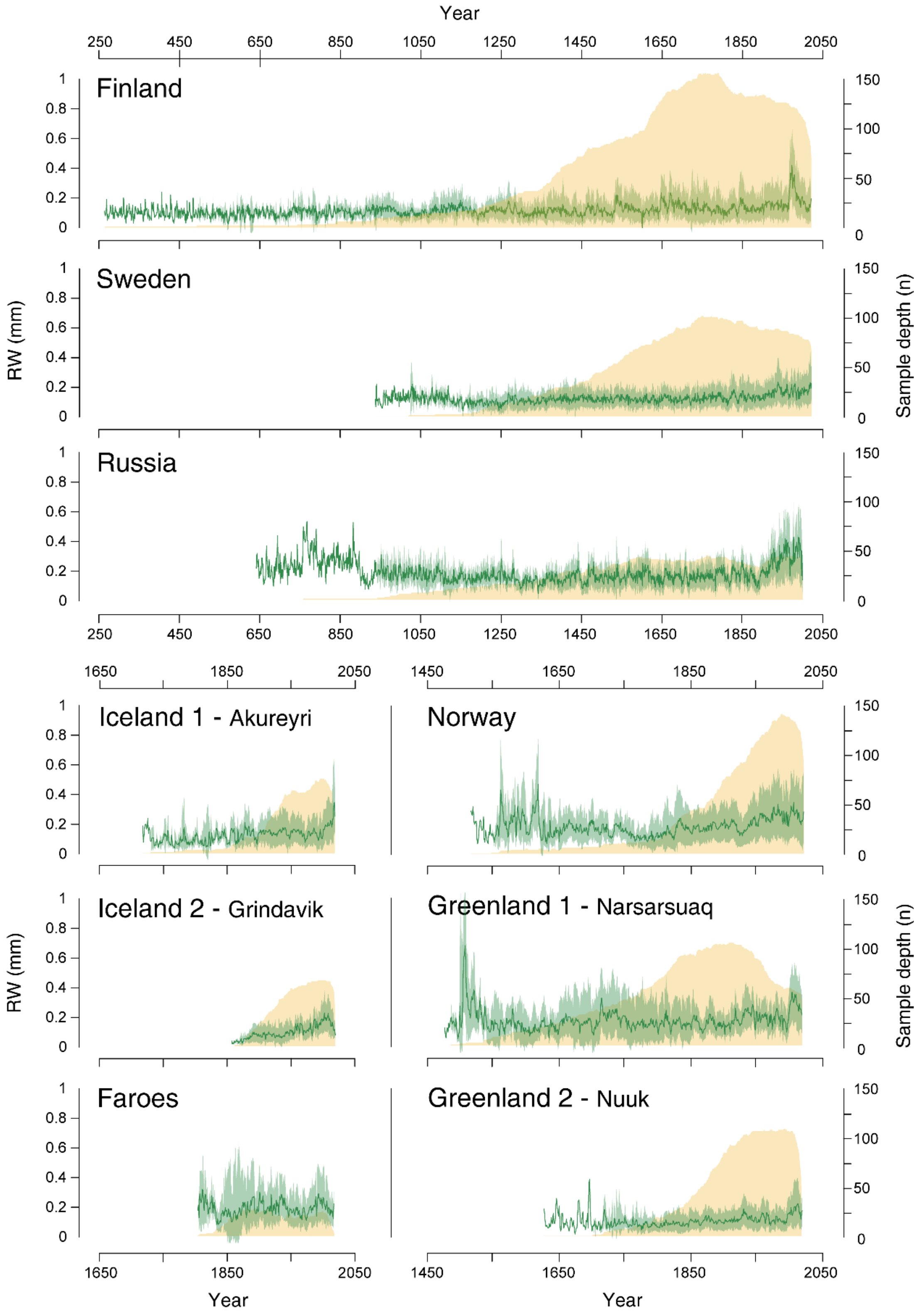

Table S2. List of the ten oldest individuals collected at each site. When the ring-width series has been crossdated, dating occurs; otherwise, the number refers to ring counting. The last row reports the starting and ending calendar years together with the length of the mean chronology.

Finnland - Kevo
69.42N 27.05E

Code	Dating	Years
kev046ad	260 - 1906	1647
kev027md	498 - 1732	1235
kev023ad	840 - 1884	1045
kev029ad	936 - 1965	1030
kev164ad	981 - 1996	1016
kev158aa	1031 - 2021	991
kev037md	494 - 1469	976
kev031aa	1054 - 2021	968
kev048aa	1081 - 2021	941
kev089aa	1206 - 2021	816
Mean Chronology	494-2021	1527

Sweden - Abisko
68.21N 18.59E

Code	Dating	Years
abi202md	845 - 2021	1176
tor079aa	1014 - 2021	1008
abi132aa	1086 - 2021	936
abi213aa	1178 - 2021	844
abi303aa	1199 - 2021	823
abi072aa	1200 - 2016	817
abi116ad	938 - 1752	815
abi158aa	1207 - 2021	815
abi180aa	1215 - 2021	808
abi205aa	1288 - 2021	734
Mean Chronology	845-2021	1176

Norway - Finse
60.35N 7.26W

Code	Dating	Years
ral146ad	1547-1965	419
ral063aa	1655-2019	365
ral008md	1686-1993	308
ral176aa	1745-2020	276
ral028ad	1516-1790	275
ral153aa	1754-2021	268
ral152ad	1771-2012	242
ral085aa	1783-2020	238
ral022ad	1638-1862	225
ral117aa	1801-2021	221
Mean Chronology	1516-2021	505

Greenland 1 - Narsarsuaq
61.09N 45.23W

Code	Dating	Years
meh185aa	1511-2019	509
hnd010bm	1581-2018	438
lnd4416m	378	378
olejc39a	1669-2015	347
olejc38a	1662-2002	341
meh187aa	1697-2017	321
meh220aa	315	315
hnd002ba	1602-1912	311
meh177am	1710-2019	310
hnd015am	1460-1768	309
Mean Chronology	1476-2020	544

Greenland 2 - Nuuk
64.11N 51.36W

Code	Dating	Years
nuu091aa	1715-2018	304
nuu114aa	1732-2017	286
nuu037ba	1759-2018	260
nuu099aa	1763-2017	255
nuu013aa	1791-2018	228
nuu062aa	1793-2018	226
nuu075aa	1792-2016	225
nuu100ab	1795-2018	224
nuu059aa	1794-2015	222
nuu037aa	1798-2018	221
Mean Chronology	1627-2018	391

Iceland 1/2 - Akureyri/Grindavik
65.43N 17.58W *63.52N 22.29W*

Code	Dating	Years
aku030aa	1731-2017	287
aku038ad	1718-1923	206
aku098am	185	185
lav002aa	1857-2019	163
aku062ad	160	160
aku065aa	1857-2016	160
aku021am	1864-2019	156
lav016aa	1870-2018	149
aku089am	1871-2017	147
lav046aa	1878-2019	142
Mean Chronology	1. 1718-2019<br>2. 1857-2019	301<br>162

Russia - Chernaya
66.49N 65.33E

Code	Dating	Years
jn0791	1160-1999	840
jn1081	988-1770	783
jn0751	1143-1887	745
jn0684	758-1438	681
jn102x	1343-1999	657
jn059x	1355-1999	645
jn1071	1080-1706	627
jn1301	996-1620	625
jn096a	1393-1999	607
jn062x	1400-1999	600
Mean Chronology	641-1999	1358

Faroe Islands - Svinoy
62.15N 6.21W

Code	Dating	Years
svi013aa	1864-2012	149
faemu16a	1841-1980	140
faemu09a	1892-2013	122
svi067aa	1897-2013	117
faemu03a	1885-1991	107
svi023aa	1914-2006	93
svi078ad	1885-1977	93
faemu01m	88	88
svi022aa	1930-2017	88
svi037aa	1932-2019	88
Mean Chronology	1818-2017	199
